# Healthcare workers’ compliance with the catheter associated urinary tract infection prevention guidelines: an observational study in Yemen

**DOI:** 10.1186/s13756-023-01352-7

**Published:** 2023-12-10

**Authors:** Khaled Mohammed Al-Sayaghi, Talal Ali Hussein Alqalah, Sameer Abdulmalik Alkubati, Sultan Abdulwadoud Alshoabi, Mohammed Alsabri, Gamil Ghaleb Alrubaiee, Mokhtar Abdo Almoliky, Khalil A. Saleh, Anas Khaled Al-Sayaghi, Rami A. Elshatarat, Zyad T. Saleh, Ahmad Mahmoud Saleh, Hassanat Ramadan Abdel-Aziz

**Affiliations:** 1 Department of Medical Surgical Nursing, College of Nursing, Taibah University, P.O. Box: 344, Al-Madinah Al‐Munawarah, 42353 Saudi Arabia; 2 Nursing Division, Faculty of Medicine and Health Sciences, Sana’a University, Sana’a, Yemen; 3 Department of Medical Surgical Nursing, College of Nursing, University of Ha’il, Ha’il City, Saudi Arabia; 4 Department of Nursing, Faculty of Medicine and Health Sciences, Hodeida University, Hodeida, Yemen; 5 Department of Diagnostic Radiology Technology, College of Applied Medical Sciences, Taibah University, Al-Madinah Al-Munawwarah, Saudi Arabia; 6 Pediatric Emergency Department, BronxCare Hospital, Bronx, USA; 7 Emergency Department, Al Thawra Modern General Hospital (TMGH), Sana’a City, Yemen; 8 Department of Community Health Nursing, College of Nursing, University of Ha’il, Ha’il City, Saudi Arabia; 9 Department of Community Health and Nutrition, Al-Razi University, Sana’a, Yemen; 10 College of Medicine and Health Sciences, Taiz University, Taiz, Yemen; 11 Faculty of Medicine, University of Alexandria, Alexandria, Egypt; 12 Department of Clinical Nursing, School of Nursing, The University of Jordan, Amman, Jordan; 13 Department of Nursing, College of Applied Medical Sciences, Prince Sattam Bin Abdulaziz University, Al-Kharj, Saudi Arabia

**Keywords:** Catheter associated urinary tract infection, Compliance, Healthcare workers, Nurses, Physicians, Prevention guidelines, Sana’a, Yemen

## Abstract

**Background:**

Catheter-associated urinary tract infection is a global problem but it can be prevented with the appropriate implementation of evidence-based guidelines. This study was conducted to assess the level of compliance of healthcare workers with the catheter-associated urinary tract infection prevention guidelines during the insertion of a urinary catheter.

**Methods:**

An observational study using a descriptive cross-sectional design was conducted at Sana’a City hospitals, Yemen. All the nurses and physicians from the governmental, teaching, and private hospitals were eligible to participate in the study. The data collection was performed through convenience sampling from March 2020 to December 2020, using a structured observational checklist prepared specifically for this study.

**Results:**

The majority of the urinary catheter insertions were performed by nurses. There were no written policy or procedures for an urinary catheter insertion and no in-service education or training departments in the majority of the hospitals. The overall mean score of compliance was 7.31 of 10. About 71% of the healthcare workers had a high or acceptable level of compliance and 29% had an unsafe level of compliance. Compliance was low for maintaining aseptic technique throughout the insertion procedure, using a single use packet of lubricant jelly, performing hand hygiene immediately before insertion, and securing the urinary catheter once inserted. Factors affecting the healthcare workers compliance were gender, the working ward/unit of the healthcare workers, the availability of a written policy/procedure and a department or unit for in-service education.

**Conclusion:**

Yemeni healthcare workers’ overall compliance was acceptable but it was unsafe in several critical measures. There is an urgent need for developing, implementing, and monitoring national guidelines and institutional policy and procedures for catheter-associated urinary tract infection prevention. Periodical in-service education and training programs and adequate access to the necessary materials and supplies are paramount.

## Background

Globally, healthcare associated infections (HAIs) are a persistent danger to patient safety. In 2011, HAIs affected 3.2 million patients in European hospitals with an estimated prevalence of 5.7% [1]. A major risk factor of HAIs is the use of invasive devices (e.g., vascular catheter, artificial airway, and urinary catheter) [1, 2]. Urinary catheterization occurs frequently and an indwelling urinary catheter (IUC) is the most prevalent indwelling device used in healthcare facilities [2, 3]. It is an important and necessary procedure for patient management in clinical settings. Approximately 100 million IUCs are used annually in the world and 16–33% of all [2, 4] hospitalized patients undergo urinary catheterization at least once during their hospitalization [5]. A IUC has many infectious and non-infectious complications, including catheter associated urinary tract infection (CAUTI), mechanical trauma, nonbacterial urethral inflammation, purulent urethritis, urethral strictures, prostatitis, and bladder urolithiasis [3].

CAUTI represents the most prevalent and costly complication associated with the use of IUC [6]. According to the National Healthcare Safety Network (NHSN), CAUTI is defined as the presence of an IUC for at least 2 days with fever and bacteriuria [2]. The source of microorganisms causing CAUTI can be endogenous, from patient’s meatal, vaginal, or rectal colonization, or exogenous, such as contaminated equipment or the hands of the healthcare workers (HCWs) [7]. The microorganisms can enter the urinary tract either by the catheter intraluminal route (internal ascension) from a contaminated urine collection bag or junction between the catheter and drainage tube, or by the catheter extraluminal route (external ascension), through movement along the external surfaces of the IUC in the periurethral mucous sheath, such as during catheter insertion without using aseptic technique [8, 9].

CAUTI is the most prevalent type of HAIs globally [9, 10], and accounts for 30-40% of all HAIs [5, 10]. Internationally, the mean CAUTI incidence is 5.07 per 1,000 catheter days in 703 ICUs in 50 countries [11]. Regionally, the CAUTI incidence was 3.2 per 1,000 catheter days [12]. In Yemen, as in the other low-income countries, the true impact of the CAUTI and the magnitude of the problem remains unidentified due to the weakness or absence of surveillance systems [13]. CAUTI represents a challenge to patient safety and the quality of the healthcare [14]. CAUTI is the largest cause of bacteremia in hospitalized patients [5, 12]. CAUTI increases bacterial resistance [12], length of hospital stay, morbidity, mortality, and healthcare costs [15]. Annually, an estimated 500,000 CAUTI events occur in the United States (US), with 13,000 deaths and $424 to $451 million direct healthcare cost [5, 13, 16]. CAUTI has significant clinical and economic consequences for patients, families, community, HCWs, and the healthcare services [17, 18].

CAUTI is an avoidable iatrogenic healthcare problem [13, 19] and its prevention should be an important goal of the infection prevention initiatives and programs at healthcare facilities. CAUTI preventive strategies are known and recommended in several evidence-based clinical guidelines [3, 6]. The strategies include inserting the UC only when appropriate, aseptic insertion technique, proper maintenance, and timely removal of the IUC [17, 20, 21]. Implementation of the recommendations results in a significant reduction in CAUTI rates [4, 16]. Poor adherence to the evidence-based CAUTI prevention measures contributes to the development of CAUTI and deters the great effort to mitigate iatrogenic infections.

Knowing which recommended CAUTI preventive measures are currently used and to what extent supports the identification of gaps in clinical practice and should be the first step for planning interventions and improving infection prevention efforts and patient safety. However, there is a shortage of studies regarding whether or not the preventive practices recommended by the guidelines are being applied in the healthcare facilities [22]. Due to a lack of similar studies locally and regionally, this study was conducted to assess the level of Yemeni HCWs compliance with the CAUTI prevention guidelines during the insertion of the urinary catheter (UC).

## Methods

### Study design

A quantitative observational (non-active approach) research was performed using a descriptive cross-sectional design.

### Population and settings

All the HCWs (nurses and physicians) in the hospitals of Sana’a city (Capital of Yemen) were eligible to participate in the study. Based on an estimated population size of 20,000 HCWs, a power level of 0.95, a response rate of 50%, and a margin of error of 0.05, a sample size of 377 HCWs was considered sufficient. A convenient sample of 403 nurses and physicians were approached, of whom 375 agreed to participate, resulting in a response rate of 93.1%. The HCWs were from all the governmental and teaching hospitals and from 3 large private hospitals in Sana’a city, Yemen.

### Inclusion and exclusion criteria

HCWs who work in adult inpatient medical or surgical wards, intensive care units (ICUs), and emergency departments (EDs) and perform urinary catheterization and who agreed to participate were included in the study. The HCWs who worked in the pediatric or neonatal units, labor and operating rooms or who refused to participate in the study were excluded.

### Data collection tool

The observation of the UC insertion was conducted using a structured observational checklist prepared specifically for this study. The instrument (checklist) contained two parts. The first part contained 13 items related to the characteristics of the HCWs, patients, UC, and workplace. The second part of the instrument contained 10 items (Table 3) related to the best evidence-based practices for CAUTI prevention during the insertion of the UC. The 10 items were in the form of checklist and rated from 0 to 1 point (Not Done = 0, Done = 1), and the total score of compliance range from 0 to 10 points. This score is converted to percentage of the score relative to the total score. The 10-item checklist was developed by the researchers based on the practices identified in the guidelines or recommendations from agencies and professional associations [5, 7, 23–26]. A panel of 2 infection control specialists, 2 bed side nurses, and 2 physicians assessed the validity of the checklist. The tool was not translated into Arabic as all the observers could speak English.

### Data collection

The data collection was performed through convenience sampling from March 2020 to December 2020, by observers who received educational sessions about the best evidence-based practices for CAUTI prevention and how to use the observation checklist. The observers were intern nurses (collected data during their internship rotations in each department) or staff nurses. Data collection was performed during the three shifts by direct observation of the catheter inserters’ compliance to the CAUTI prevention guidelines. The patient same-sex observers used the structured observational checklist to observe and document the UC insertion without participation. During the observation, for each item in the checklist if the inserter performed an action consistent with the CAUTI prevention guidelines, the observer documented “done”; if the inserter demonstrated an action inconsistent with CAUTI prevention guidelines, missed it, or failed to apply it the observer documented “not done”. One observational sheet was filled out for each inserter, regardless of the number of UC insertions they performed. Once a HCW had been initially observed and agreed to participate in the study, he was included in the study sample. Subsequent UC insertions by the same HCW were not observed or counted as new participations within the study sample. This practice was adopted to mitigate potential observation bias, as HCWs were already aware of being under observation. Furthermore, if an inserter was counted multiple times, the data would be skewed depending on the inserter’s performance level.

### Ethical considerations

Ethical approval was obtained from the Research Ethical committee of Al-Razi University (022/FORMS/2020). Permission was obtained from the hospitals’ administrations and units’ managers where the study was conducted. Informed consent was obtained from all the participants (inserters). The covert observation was performed in a way that the catheter inserter did not note the presence of the observer to decrease the Hawthorne effect [27, 28]. After the completion of each observation, the inserter was approached in complete secrecy and was informed of the truth and the objectives of study. Inserters were informed that their participation were voluntarily, and they could withdraw from the study without any consequences. They were assured that their anonymity and confidentiality will be maintained throughout the study. If the catheter inserter agreed to participate in the study, he/she was considered a participant in the study. If the catheter inserter did not agree to participate in the study, the observation checklist was excluded from the study (28 participants were excluded). Participants were identified by code to preserve their anonymity. No names or other identification data were collected.

### Statistical analysis

Data analysis was done using the Statistical Package for Social Sciences (SPSS) version 25. Compliance with individual items in the checklist is presented as frequency and percentage. The compliance score was calculated for each participant. A test of normality indicated a normal distribution of the score. The compliance score was converted to a dichotomous variable as follows: a score < 50th percentile (< 7.0) was classified as unsafe compliance, a score between the 50th and 75th percentiles (7.0–8.0) was as classified acceptable compliance, and a compliance score > 75th percentile (> 8.0) was classified as high compliance. This scoring was used based on previous observational studies conducted for similar purposes [29]. The relationships between the study variables and the compliance score were measured by using the independent t-test, and the one-way analysis of variance (ANOVA). The factors that were significant in the univariate analysis were entered in the multiple linear regression analysis (enter technique) to determine the factors affecting the HCWs compliance. The accepted level of significance was below 0.05 (p < 0.05).

## Results

A total of 403 urinary catheterization procedures of 403 patients were recorded during a period of 9 months. In total, 375 UC insertion observations were analyzed and 28 observations were excluded from data analysis because the catheter inserter did not agree to participate in the study. The majority of the observed urinary catheterization procedures were performed by the nurses (76.8%). Male HCWs performed the majority of the observed catheterization procedures (59.2%). Less than half (41%) of the observed urinary catheterization procedures were in governmental hospitals, 35.5% in private hospitals, and 24% in teaching hospitals. More than a third (36%) of the observed catheterizations were in the medical wards, 32% in ICUs, 23.7% in surgical wards, and 8.8% in EDs. The majority of the wards (82.7%) where the catheterization procedure was performed do not have any written policy or procedure for the UC insertion and the majority (63.5%) did not have a department or unit for continuous education (Table 1).

**Table 1:** Compliance of the HCWs with each individual item of the CAUTI prevention guidelines (n = 375)

Guidelines Recommendations	Done		Not Done	
	n	%	n	%
Perform hand hygiene immediately before insertion	252	67.2	123	32.8
Use sterile gloves, drapes and sponges	318	84.8	57	15.2
Clean urethral meatus with antiseptic solution before insertion	328	87.5	47	12.5
Use a single use packet of lubricant jelly	186	50.4	189	49.6
Use small size catheter to minimize trauma	292	77.9	83	22.1
Maintain aseptic technique throughout the insertion procedure	154	41.1	221	58.9
Secure catheter to leg with tape once inserted	254	67.7	121	32.3
Maintain the urine bag below the level of the patient and off the floor	337	89.9	38	10.1
Maintain unobstructed urine flow (make sure no kinking in the catheter once inserted)	323	86.1	52	13.9
Perform hand hygiene immediately after insertion	299	79.7	76	20.3

Table 2 displays that 63.7% of the patients who received the UCs were male, with a mean age of 44.8 years old. The most frequent patient disorder was cardiac disorders (17.9%), followed by neurological and renal disorders (14.9% for each). The most frequent indication for urinary catheterization was the need for urine output monitoring (36%), followed by an unconscious state (27.5%), and perioperative (16.8%). Of note, a retention UC (IUC with a double lumen and balloon) was the most frequent (95.7%) type used in Sana’a hospitals. Silicon catheters were the most frequently used (49.3%), followed by Latex catheters (39.2%). The catheter sizes from 16 to 18 fr were mostly used (55.3%), followed by sizes from 12 to 14 fr (32.3%).

**Table 2:** Characteristics of the UC inserters and hospitals

Characters of Catheter Inserters and Hospitals	n (375)	%
**Job title of Catheter Inserter**		
Nurse	288	76.8
Physician	87	23.2
**Gender of Catheter Inserter**		
Male	222	59.2
Female	153	40.8
**Type of Hospital**		
Governmental	152	40.5
Teaching	90	24
Private	133	35.5
**Ward (department)**		
Medical Wards	133	35.5
Surgical Wards	89	23.7
ICUs	120	32.0
EDs	33	8.8
**Presences of a written protocol/ policy/procedure**		
Yes	65	17.3
No	310	82.7
**Presences of continuous education department/unit**		
Yes	127	36.5
No	238	63.5

EDs: Emergency Departments; ICUs: Intensive Care Units

Table 3 reveals the HCWs’ compliance level was high (≥ 85%) in four items of the CAUTI prevention guidelines, including maintaining the urine bag below the level of the patient and off the floor, cleaning the urethral meatus with antiseptic solution before catheter insertion, maintaining an unobstructed urine flow, and using sterile gloves, drapes and sponges. The HCWs’ compliance level with using a small size catheter and performing hand hygiene immediately after insertion was acceptable. However, it is disturbing that the compliance was below the acceptable level (< 70%) in the remaining four items, including the performing of hand hygiene immediately before UC insertion, using a single use packet of lubricant jelly, securing the UC to a leg with tape once inserted, and maintaining the aseptic technique throughout the UC insertion procedure. It is alarming that the lowest compliance level (41%) was observed for an important item related to maintaining aseptic technique throughout the UC insertion procedure.

**Table 3:** Characteristics of the patients and UCs

Characters of Patients and Catheters	n (375)	%
**Patient’s Gender**		
Male	239	63.7
Female	136	36.3
**Patient age (years)**		
12- <18	19	5.1
18–30	77	20.5
> 30–40	77	20.5
> 40–50	63	16.8
> 50–60	64	17.1
> 60	75	20
**Patient Diagnosis**		
Cardiac disorders	67	17.9
Respiratory disorders	34	9.1
Neurologic disorders	56	14.9
Renal disorders	56	14.9
Urological disorders	47	12.5
Endocrine disorders	18	4.8
Oncology (Cancer)	22	5.9
GIT and Hepatobiliary disorders	32	8.5
Musculoskeletal disorders	26	6.9
Other (obstetric, gynecology disorder … etc.)	17	4.5
**Indications/reasons for Catheterization**		
Patient unconscious	103	27.5
For urine output monitoring	135	36
Perioperative	63	16.8
Patient on bed rest or bedridden	50	13.3
Others	24	6.4
**Type of Catheter**:		
Retention catheter (IUC with double lumen and balloon)	359	95.7
Straight catheters (a single-lumen without balloon)	16	4.3
**Catheter Material**		
Silicon	185	49.3
Latex	147	39.2
Other (Plastic, Rubber)	43	11.5
**Catheter Size**		
6–10	19	5.1
12–14	121	32.3
16–18	208	55.5
≥ 20	27	7.2

GIT: Gastrointestinal Tract; IUC: Indwelling Urinary Catheter

The overall mean score of compliance was 7.31 out of 10. Less than quarter of the observed HCWs (22.7%) were categorized in the high level of compliance (> 8 out of 10) and 48.5% categorized in the acceptable level (7 to 8 out of 10). Of note, 29% of the observed HCWs were categorized in the unsafe level of compliance (< 7 out of 10). As shown in Table 4, the female HCWs had significantly higher compliance with the CAUTI prevention guidelines compared to the male HCWs (p = 0.011). The HCWs working in the ICUs demonstrated significantly higher level of compliance than the HCWs in the other wards (p = 0.021). HCWs working in the wards that have a written policy for UC insertion and maintenance, and the HCWs working in the hospitals with an in-service education department had significantly higher compliance than the HCWs working in the wards without a written policy or a continuous in-service education department (p = < 0.001).

**Table 4:** Compliance scores according to the characteristics of the inserter, hospitals, and catheters

	n (%)	Compliance Score		*P*
		Score from 10 Mean ± SD	Score from 100 Mean ± SD	
**Total Sample**	375 (100)	7.31 ± 1.73	73.15 ± 17.33	
**Job title of Catheter Inserter**				
Nurse	288(76.8)	7.28 ± 1.78	72.81 ± 17.79	0.498
Physician	87(23.2)	7.43 ± 1.57	74.25 ± 15.75	
**Gender of Catheter Inserter**				
Male	222(59.2)	7.13 ± 1.82	71.26 ± 18.23	0.011
Female	153(40.8)	7.59 ± 1.55	75.88 ± 15.58	
**Ward (department)**				
Medical Wards	133 (35.5)	7.20 ± 1.77	72.03 ± 17.74	0.021
Surgical Wards	89 (23.7)	7.12 ± 1.81	71.24 ± 18.08	
ICUs	120 (32.0)	7.70 ± 1.80	77.00 ± 16.22	
EDs	33 (8.8)	6.88 ± 1.56	68.79 ± 15.56	
**Is there a written policy/procedure for catheter insertion and maintenance?**				
Yes	65 (17.3)	8.02 ± 1.58	80.15 ± 15.76	< 0.001
No	310 (82.7)	7.17 ± 1.73	71.68 ± 17.30	
**Type of Hospital**				
Governmental	152 (40.5)	7.16 ± 1.87	71.58 ± 18.70	0.059
Teaching	90 (24)	7.16 ± 1.51	71.56 ± 15.06	
Private	133 (35.5)	7.60 ± 1.69	76.02 ± 16.87	
**Is there a in-service education department in the hospital?**				
Yes	127 (36.5)	8.06 ± 1.50	80.58 ± 14.99	< 0.001
No	238 (63.5)	6.89 ± 1.72	68.87 ± 17.16	
**Type of Catheter**:				
Retention catheter	359 (95.7)	7.32 ± 1.75	73.23 ± 17.51	0.655
Straight catheters)	16 (4.3)	7.12 ± 1.25	71.25 ± 12.58	

EDs: Emergency Departments; ICUs: Intensive Care Units

In the multivariate analysis (Table 5), the females had a significantly higher level of compliance compared to the males (p = 0.019), and the HCWs in the ICUs compared to the group working in the EDs (p = 0.006), compliance was significantly higher in wards with a written policy for catheter insertion and maintenance (p = 0.005) and in the group with a continuous education department in their hospitals (p < 0.001). The total model was significant (p = 0.001) and the data fit the model. The model explains 15% of the variance of the compliance score. There was no multicollinearity.

**Table 5:** Factors influencing compliance with CAUTI prevention guidelines in multivariate analysis

	B	SE	Beta	95% CI of B	*p* value
**Gender of Catheter Inserter**					
Female	0.40	0.17	0.11	5.42–6.57	0.019
Male	Reference				
**Ward (department)**					
ICUs	0.57	0.21	0.15	0.16–0.98	0.006
EDs	Reference				
**Presence of written policy/procedure for catheter insertion and maintenance**	0.61	0.22	0.13	0.17–1.05	0.005
**Presence of continuous education department in the hospital**	1.03	0.18	0.28	0.69–1.38	< 0.001

EDs: Emergency Departments; ICUs: Intensive Care Units

## Discussion

This study is to our knowledge, the first national and regional observational study of CAUTI prevention practices of physicians and nurses. This study provides novel insight into CAUTI prevention area and identifies the gap between the evidence-based recommendations and the current HCWs practices for CAUTI prevention. The overall mean score of compliance of Yemeni HCWs barely reached the acceptable level (7.31 out of 10). The compliance to several critical CAUTI prevention guideline measures was low, which decreased the general compliance of Yemeni HCWs. This level of Yemeni HCWs compliance may be linked, as the study results indicated, to the lack of a written policy or procedure for the UC insertion and the absence of a department/unit for the in-service education in the majority of the hospitals. We hypothesize that the Yemeni HCWs compliance level with CAUTI prevention guidelines can also be explained by the current state of healthcare services in the Yemen, including understaffing, inadequate working conditions, frequent shortages of necessary supplies, and overcrowded hospitals. However, these factors were not examined in this study. This deterioration in healthcare services reflects the difficult living conditions, as well as stress and frustrations in the daily life of Yemeni people. The dominant political violence, instability, and deteriorating economic conditions of the country are the primary causes underlying all the above deterioration.

Of note, 29% of the observed Yemeni HCWs were in the unsafe level of compliance (scored < 7 out of 10). Interestingly, this finding is lower than reported in a self-reported study by Algarni et al. (2019) who found that 83.94% of Saudi nurses have a poor level of practice with CAUTI prevention [30]. Similarly, Mong et al. (2022) [10], Conway et al. (2012) [22], Opina and Oducado (2014) [31], and Methu et al. (2019) [32] found that the implementation of guidelines for CAUTI prevention is inadequate and insufficient.

The finding of this study that the ward in which the HCW was working affect their level of compliance with the CAUTI prevention practices was in line with literature [30, 33]. The lower compliance level of the HCWs in the EDs could be attributed to the nature of the ED environment and certain prevalent ED practices. The infection prevention practices are usually overlooked in the fast-paced environment where life-threatening conditions take priority [34]. The findings of our study that the female HCWs had a significantly higher compliance level with the CAUTI prevention practices than their male colleagues was in contrast to an earlier study, concluding that the gender of the HCW who inserted the UC does not affect the compliance level [10, 33].

This study found that, in line with Oman et al. (2012), the presence of a policy positively impacted the HCWs’ compliance [35]. Policies and procedures in healthcare are aimed to standardize practice, to incorporate evidence-based practices, and to attain regulatory compliance. Our finding that the presence of an in-service education department/unit in the hospital positively affect the HCWs’ compliance was supported by Saint et al. (2016), who concluded that hospitals with a residency training program were > 4 times as likely to adopt a policy than hospitals without residency training programs [20]. Similarly, Oman et al. (2012) reported that training positively impact nurses’ compliance and CAUTI rates [35]. Medical and nursing education and healthcare services suffered extensively from a lack of essential resources due to the conflict in Yemen. A significant proportion of the HCWs studied, graduated, and worked during the internal conflict. In most Yemeni hospitals, quality projects and in-service education programs have been suspended or terminated. Infection prevention becomes a low priority in a conflict setting.

Although many CAUTI preventive measures had a good compliance rate, no item had 100% compliance. About one third (32.8%) and one fifth (20.3%) of the observed HCWs did not perform hand hygiene before and after catheter insertion, respectively. This finding is consistent with the other studies reported that from 10 to 40% of the nurses did not perform hand washing prior to UC insertion [30–33, 36, 37]. While the Yemeni HCWs compliance was acceptable in using a small size catheter (77.9%), an unsafe level of compliance was observed with the use of single pack lubricant jelly (50.4%) and securing the UC once inserted (67.7%). This finding contradicted the finding of other studies that reported 84% of nurses used a single pack lubricant jelly [36] and 30% secured the UC once inserted [32].

Although maintenance of aseptic technique throughout the UC insertion procedure is a critical item in the CAUTI prevention guidelines, the lowest compliance level (41.1%) was observed for this important item. This finding contradicted the finding of several studies that reported 74–95% compliance rate with aseptic technique during the UC insertion [1, 37, 38].

Of note, 87.5% of Yemeni HCWs clean the urethral meatus with an antiseptic before UC insertion and 84.8% use sterile gloves in UC insertion. This compliance level is lower than that reported in previous study that 97% of nurses always use sterile gloves [37]. The majority of the Yemeni HCWs (> 85%) followed good practices in terms of keeping the tubing and collecting bag free from kinking and below the level of the patient’ bladder. Studies reported that these items were complied with at all times, which is congruent with our study [14, 33, 39].

The low adherence of Yemeni HCWs to some preventive measures could be attributed to the inconsistent or inconvenient locations of hand sanitizers, limited resources and lack of supplies, such as the hand hygiene preparations, sterile gloves, different sizes of UC, single-use lubricant gel and a catheter-securing device in most hospitals [33, 34, 38]. This is one of the catastrophic consequences of the internal armed conflict during the last eight years in Yemen.

Compliance with isolated CAUTI preventive measures is not effective, prevention of CAUTI requires that all the HCWs are fully compliant to all the recommended preventive measures collectively and simultaneously. This study suggests that there is definite room for improvement in Yemeni HCWs practices. There is urgent need for developing, implementing, and monitoring national guidelines and an institutional policy and procedures for CAUTI prevention to reduce the gap between the evidence-based recommendations and the HCWs practices. To comply fully with the CAUTI preventive measures requires the provision of the necessary materials and supplies; however, the availability of the materials and supplies is not sufficient if they are not used correctly. There must be periodical in-service education and training programs for HCWs and adequate access to the necessary materials and supplies.

### Limitations

Firstly, we focused on the HCWs compliance with the CAUTI prevention guidelines during UC insertion procedure only. The other UC care procedures were beyond the scope of the study. Secondly, the sample of hospitals represent Sana’a city (capital of Yemen). The situation in the hospitals outside Sana’a city may be worse. Additional studies integrating the assessment of the actual practice and auditing the impact on the CAUTI incidence are recommended.

### Conclusions

Yemeni HCWs’ overall compliance was acceptable but it was unsafe in several critical measures, such as aseptic technique, using a single use packet of lubricant jelly, performing hand hygiene before insertion, and securing the UC once inserted. Several factors significantly affect the HCWs compliance with the CAUTI prevention guidelines, including gender and the working ward/unit of the HCWs, and the availability of a written policy or procedure and a department or unit for continuous in-service education.

## Data Availability

The raw data are available from the corresponding author on reasonable request.

## Abbreviations

ANOVA: One-Way Analysis of Variance
CAUTI: Catheter Associated Urinary Tract Infection
EDs: Emergency departments
HAIs: Healthcare Associated Infections
HCWs: Healthcare Workers
IUC: Indwelling Urinary Catheter
ICUs: Intensive Care Units
NHSN: National Healthcare Safety Network
SPSS: Statistical Packages for Social Sciences
UC: Urinary Catheter
US: United States
